# A Somatically Diversified Defense Factor, FREP3, Is a Determinant of Snail Resistance to Schistosome Infection

**DOI:** 10.1371/journal.pntd.0001591

**Published:** 2012-03-27

**Authors:** Patrick C. Hanington, Michelle A. Forys, Eric S. Loker

**Affiliations:** 1 Environmental Health Sciences, School of Public Health, University of Alberta, Edmonton, Alberta, Canada; 2 Center for Evolutionary and Theoretical Immunology, Department of Biology, University of New Mexico, Albuquerque, New Mexico, United States of America; Biomedical Research Institute, United States of America

## Abstract

Schistosomiasis, a neglected tropical disease, owes its continued success to freshwater snails that support production of prolific numbers of human-infective cercariae. Encounters between schistosomes and snails do not always result in the snail becoming infected, in part because snails can mount immune responses that prevent schistosome development. Fibrinogen-related protein 3 (FREP3) has been previously associated with snail defense against digenetic trematode infection. It is a member of a large family of immune molecules with a unique structure consisting of one or two immunoglobulin superfamily domains connected to a fibrinogen domain; to date fibrinogen containing proteins with this arrangement are found only in gastropod molluscs. Furthermore, specific gastropod FREPs have been shown to undergo somatic diversification. Here we demonstrate that siRNA mediated knockdown of *FREP3* results in a phenotypic loss of resistance to *Schistosoma mansoni* infection in 15 of 70 (21.4%) snails of the resistant BS-90 strain of *Biomphalaria glabrata*. In contrast, none of the 64 control BS-90 snails receiving a GFP siRNA construct and then exposed to *S. mansoni* became infected. Furthermore, resistance to *S. mansoni* was overcome in 22 of 48 snails (46%) by pre-exposure to another digenetic trematode, *Echinostoma paraensei*. Loss of resistance in this case was shown by microarray analysis to be associated with strong down-regulation of FREP3, and other candidate immune molecules. Although many factors are certainly involved in snail defense from trematode infection, this study identifies for the first time the involvement of a specific snail gene, *FREP3*, in the phenotype of resistance to the medically important parasite, *S. mansoni*. The results have implications for revealing the underlying mechanisms involved in dictating the range of snail strains used by *S. mansoni*, and, more generally, for better understanding the phenomena of host specificity and host switching. It also highlights the role of a diversified invertebrate immune molecule in defense against a human pathogen. It suggests new lines of investigation for understanding how susceptibility of snails in areas endemic for *S. mansoni* could be manipulated and diminished.

## Author Summary

Schistosomiasis, a neglected tropical disease, owes its continued success to freshwater snails that support production of prolific numbers of human-infective cercariae. Encounters between schistosomes and snails do not always result in the snail becoming infected, in part because snails can mount immune responses that prevent schistosome development. Understanding the factors important for snail resistance to schistosome infection will facilitate new lines of investigation to 1) understand the underlying basis of compatibility between schistosomes and snails in endemic areas and how this affects transmission dynamics and control efforts; and 2) to reveal ways to manipulate natural snail populations to enhance their resistance to schistosome infections. Here, we present the first evidence that a snail immune molecule, fibrinogen related protein 3 (FREP3), is important for successful defense against schistosome infections in *Biomphalaria* snails. In addition, we demonstrate that FREP3 is a target suppressed by trematode parasites to facilitate their establishment within the snail.

## Introduction

Schistosomiasis is one of the world's most tenacious neglected tropical diseases, infecting an estimated 207 million people, mostly children [1]. The persistence of schistosome parasites stems in part from their use of freshwater snails for their larval development and transmission. Snails are often abundant and difficult to control, and it is in snails that the cercariae infective to humans are produced in prolific numbers. It takes only a single schistosome miracidium to establish a snail infection capable of producing hundreds of cercariae on a daily basis for months [2]. The amplification of schistosomes that occurs within snails creates a reoccurring problem for control efforts and is a significant obstacle for sustained prevention. It highlights the importance of understanding the dynamics of schistosome infections in snails and is the reasoning behind studies focused on characterizing the mechanistic basis for snail resistance to schistosome infection. If we could understand the underlying factors that enable snails to resist schistosome infection, then we could better understand the basis of compatibility in field snails. The level of compatibility exhibited will directly influence both transmission dynamics and control efforts. We could also potentially exploit resistance to favor development of more sustainable control strategies that go beyond today's largely one-dimensional control programs that depend primarily on treatment of infected people with praziquantel [3].

Not all snails are created equal: some are susceptible and some resistant to schistosome infection. Resistance is genetically controlled and affects immunological factors [4], [5] that vary among snail species, strains or age categories. For example, the human parasite *Schistosoma mansoni* infects only certain species of *Biomphalaria* (such as *B. glabrata*). Furthermore, only some strains of *B. glabrata* are compatible with this parasite. Many studies have focused on characterizing the transcriptional profiles of schistosome resistant strains compared to susceptible counterparts, and have identified a number of putative resistance-associated factors in the process [6], [7]. Amongst these molecules are the fibrinogen-related proteins (FREPs), members of a multi-gene family that undergo somatic diversification and point mutation events. FREP proteins couple together fibrinogen and immunoglobulin superfamily domains, to generate a protein that is unique as far as presently known to gastropod molluscs [8]. FREPs are capable of precipitating secretory/excretory products from digenetic trematode sporocysts [9], and binding to diversified glycoproteins produced by parasites [10]. One individual FREP, FREP3, has been singled out for further study because of its role in the snail defense response against the trematode *Echinostoma paraensei* [4]. FREP3, like other FREPs, is a lectin-like molecule that recognizes a number of monosaccharides and is able to enhance the phagocytic uptake of targets, acting as an opsonin [11]. Knockdown of FREP3 in a normally resistant snail phenotype, and subsequent challenge of those snails with *E. paraensei* resulted in a significant proportion of the snails becoming infected with *E. paraensei* [11].

Trematode infection of a snail host is achieved, in part, by evading and suppressing the snail defense response. This provides a window for establishment of infection and then preventing the immune response from interfering with parasite development. These immune-evasion strategies can be observed *in vitro* [12], and also by transcriptional analysis [7], which suggests that many of the transcripts expressed by resistant snails during successful defense are suppressed in susceptible snails that become infected [11]. Immunosuppression is especially strong following exposure to *E. paraensei*, a parasite that can alter snail hemocyte morphology and interfere with hemocyte function [12], and that can suppress the expression of important immune molecules almost immediately upon entry into the snail [7]. One of the factors we identified as being suppressed by *E. paraensei* during infection is FREP3 [11]. This observation prompted us to use, in one of the experiments described below, a protocol first employed by Lie and Heyneman [13] in which pre-exposure of schistosome-resistant snails to *E. paraensei* was used to abrogate resistance to subsequent schistosome infection. We hypothesized specifically that this treatment would interfere with FREP3 expression (and likely with expression of other immune components as well), as compared to schistosome-resistant control snails not exposed to *E. paraensei*.

In this study, we report on the results of two different manipulations undertaken with the intention of abrogating resistance to *S. mansoni* in the naturally resistant BS-90 strain of *B. glabrata*. We first examined the effects of knocking down FREP3 using RNAi on the subsequent ability of BS-90 snails to support *S. mansoni* development. Secondly, we also expressly repeated the classic experiment of Lie et al. (1977) [14], using both BS-90 snails and accompanying microarray monitoring for the first time. We first exposed BS-90 snails to radiation-attenuated miracidia of *E. paraensei*, and then assessed their resistance level to *S. mansoni* as compared to snails not pre-exposed to *E. paraensei*. Radiation-attenuated *E. paraensei* parasites do not establish long-term, proliferative infections in snails, avoiding the potential complication that persistent larvae of this species would prevent the potential development of *S. mansoni*.

It is known, however, that irradiated *E. paraensei* larvae, during their brief lifespan, exert a potent immunosuppressive effect just as do normal *E. paraensei* larvae [7], [11], [13]. Infection with *S. mansoni* of FREP3 knockdown snails and those first exposed to irradiated *E. paraensei* was also assessed by histological examination as well as by checking for shedding *S. mansoni* cercariae, which were tested for infectivity to mice. We compared the transcriptional profiles of BS-90 snails exposed only to irradiated *E. paraensei* to those exposed to irradiated *E. paraensei* and then challenged with *S. mansoni*. Our study seeks to demonstrate the involvement of a specific molecule in snail resistance to *S. mansoni* infection, and to provide a plausible natural mechanism by which trematode-mediated immunosuppression of the defense responses of a snail could facilitate infection by a parasite that it would normally successfully resist.

## Materials and Methods

### Live material

BS-90 and M-line strain *Biomphalaria glabrata* (*B.g.*) snails, and *Schistosoma mansoni* (*S.m.*) and *Echinostoma paraensei* (*E.p.*) were maintained as previously described [15].

### FREP3 knockdown

Four independent 27 nucleotide oligos were designed to specific regions of FREP3 that displayed high conservation within the known diversified FREP3 transcripts. These oligos were combined and diluted in sterile snail saline at a final total concentration of 2 µg/µl, which was then injected into snails in a 5 µl volume. BS-90 snails were separated into two groups, the first to be injected individually with FREP3-specific siRNA oligos, and the second as a control, with GFP-specific oligos [16], siRNA oligo design and injection techniques have been previously described [11]. Four hours later, all snails were exposed individually to 30 *S.m.* miracidia. Snails were collected for histology at 2, 8, 18, 21, and 28 dpe. Snails were examined for the presence of infection (presence or absence of primary and secondary sporocysts) as described above for signs of infection at 21, 28, 34, 41, 48, and 54 dpe. Snails that shed cercariae were collected for histology and the rest were dissected to look for infections.

Knockdown of FREP3 was confirmed by RT-PCR and western blot analysis both of which have been previously described [11]. Specific knockdown of FREP3 protein levels was confirmed by probing the same samples with a FREP4 specific antibody. For both Western blot analyses, 100 µg of cell free plasma was loaded into each well of an SDS acrylamide gel. FREP3 was detected using a FREP3 specific antibody, and the Western blot was developed using the Supersignal West Femto Chemiluminescent Substrate (Pierce). FREP4 was detected using a FREP4-specific antibody and the Western Blot was developed using alkaline phosphatase. Injection of siRNA oligos and challenge of both FREP3 knockdown and GFP knockdown snails with *S. mansoni* resulted in similar mortality in both groups of snails. 36% of the FREP3 knockdown snails and 31% of the GFP knockdown snails died as a result of treatment.

### Microarray confirmation of FREP3 RNAi-mediated knockdown specificity

In addition to RT-PCR and Western blot confirmation of FREP3 knockdown using FREP3-specific siRNA oligos as previously described [11], we confirmed the specificity of FREP3 knockdown using microarray analysis. BS-90 snails (4–8 mm) were injected with either FREP3 or GFP-specific siRNA oligos, and 2 hours later exposed to 30 *S.m.* miracidia. At 2 and 4 dpe, ten snails from each group were collected, RNA was extracted and then used to generate template for the microarray as previously described [15]. Ten arrays were completed. Each array was probed with template from an individual FREP3 knock-down snail labeled with Cy5 and an individual GFP knock-down snail labeled with Cy3. Hybridization, scanning, and analysis of the microarrays were previously described [7], using a significance cutoff of +/−log 1.5, and a false detection rate of 5%. Microarray results were submitted to GEO under the accession number GSE33525. The microarray revealed that indeed FREP3 expression was reduced at the transcriptional level by 2.4 fold at 2 dpe and by 5.1 fold at 4 dpe. The only other significant results from that array revealed a slight reduction in FREP13 expression by 1.2 fold at 2 dpe and 2 fold at 4 dpe and a slight up-regulation of TGFR-1 at 1.8 fold at 2 dpe and 2.6 fold at 4 dpe (**Fig. S1**). 18 other transcripts including FREP2 and FREP6 displayed slight alterations in expression however these changes were not considered statistically significant; none of these other 16 transcripts were FREPs.

### Confirmation of *S. mansoni* infectivity to a mouse from a FREP3 knock-down snail

To confirm viability of the cercariae produced from the FREP3 knock-down BS-90 snails that shed at 31 dpe we collected all cercariae produced (∼150), and exposed one mouse, using standard procedures as previously described [17]. Seven weeks post-exposure, the mouse was injected with a heparin solution and perfused by cutting the hepatic portal vein and injecting a standard RPMI medium into the heart. *S. mansoni* adult worms were collected and the liver was homogenized to collect *S. mansoni* eggs. The presence of adult worms confirmed the cercariae isolated from BS-90 snails were viable and the miracidia hatched from the eggs were also viable, being able to infect M-line *B. glabrata* snails (data not shown).

### *Echinostoma paraensei* immunosuppression and schistosome challenge

Size (4–8 mm shell diameter) matched snails were distributed into five groups: 1). BS-90 exposed to 25–30 irradiated *E.p.* miracidia at day 0 and secondarily challenged 4 days later with 15 *S.m.* miracidia, 2). BS-90 exposed to 25–30 irradiated *E.p.* only at day 0, 3). BS-90 unexposed control, 4). BS-90 exposed to only 15 *S.m.* miracidia at day 4, and 5). M-line exposed to only 15 *S.m.* miracidia at day 4 to confirm *S.m.* infectivity. For groups 2 and 3, RNA was collected at 1, 2, and 4 days post-exposure (dpe) to *E.p.* RNA was collected from groups 1, 2 and 4 at 1, 2, 4, and 8 dpe to *S.m.* and snails were collected for histology from groups 1 and 4 at 2, 8, 18 and 28 dpe to *S.m.* Snails from group 5 at 18 dpe to *S.m.* were also collected for histology. At days 18, 28, and 34 post-exposure to *S.m.*, all remaining snails were placed into large tissue culture wells with artificial spring water and examined for the presence of developing primary sporocysts in the head foot or mantle, or secondary sporocysts in the mantle or digestive gland/ovotestes. Snails that were shedding *S.m.* cercariae were collected for histology. All snails that did not shed cercariae, were individually placed in snail saline, dissected and examined with the aid of a dissecting microscope for any signs of infection (sporocysts, germ balls, cercarial embryos) which dissection of known infected snails indicates can be seen under the 40× magnification used. Irradiation of *E.p.*, and RNA extraction were previously described [15].

### Microarray analysis of resistance loss due to infection with *E. paraensei*

RNA was collected from whole snails at 2 and 4 dpe to 15 *S.m.* miracidia from groups 1 and 2 above, and was used to generate template for the microarray as previously described [15]. Each array was probed with RNA from an individual snail from the experimental group (1 from above), labeled with Cy5 and with RNA from a snail from control group 2, labeled with Cy3. There were twelve arrays in total, six from 2 dpe and six from 4 dpe, as a previous study revealed a great differentiation in transcription between these two time points [7]. Hybridization, scanning, and analysis of the microarrays were previously described [7], using a significance cutoff of +/−log 1.5, and a false detection rate of 5%. Microarray results were submitted to GEO under the accession number GSE28293.

### Histological analysis of *S. mansoni* infection

Snails were collected and placed whole into tubes containing Railliet-Henry's fixative (930 ml H_2_O, 50 ml formalin, 20 ml acetic acid, and 6 g NaCl) to both fix the tissue and dissolve the shell. Any remaining shell was removed before the tissue was transferred into 10% buffered formalin. All tissue processing, sectioning, mounting, and hematoxylin and eosin staining was performed by TriCore Reference Laboratories in Albuquerque, New Mexico. The images generated from these sections were taken using a Nikon D5000 SLR camera attached to a Zeiss Axioskop compound microscope with an MM-SLR adapter and T-mount by Martin Microscope Company.

## Results

### Abrogation of resistance to *S. mansoni* following RNAi knockdown of FREP3

**Figure 1:**
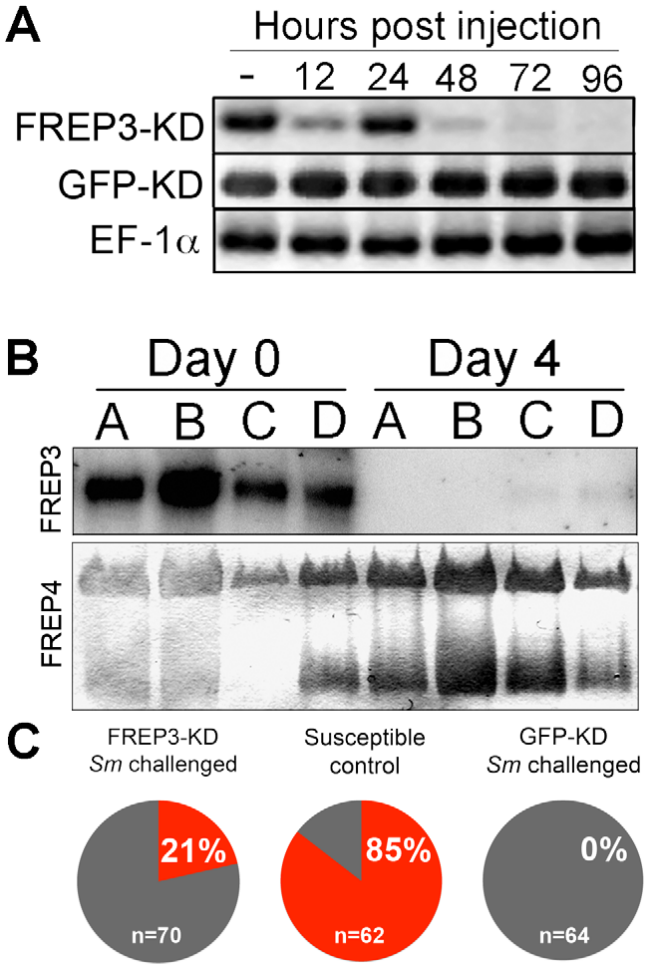
Knockdown of FREP3 reduces resistance to *S. mansoni* infection in BS-90 strain *B. glabrata*. A) RNAi knockdown of FREP3 in BS-90 *B. glabrata* snails confirmed at the transcriptional level by RT-PCR (25 cycles) over the course of 96 hours post injection. Shown are representative results from injection of FREP3-specific siRNA oligos and control GFP-specific siRNA oligos on FREP3 transcript expression. Experimental values are compared to the endogenous control elongation factor 1-α (EF-1α). B) Confirmation of protein-level knockdown of FREP3 in 4 individual BS-90 snails (A–D) before, and 4 days after injection of FREP3-specific siRNA oligos. FREP3 was visualized using a specific anti-FREP3 antibody. As a control for protein loading 100 µg of cell-free plasma was loaded into each well, and the same samples were probed for FREP4, using an anti-FREP4 antibody. FREP4 is a different FREP family member, related to FREP3, and was detectable in all individuals both before and after FREP3 knockdown. C) Percentage of BS-90 snails shedding *S. mansoni* cercariae (21%) after knockdown of FREP3, as compared to controls which were either susceptible M-line snails exposed to *S. mansoni* (85%) or BS-90 snails injected with the GFP siRNA constructs and challenged with *S. mansoni* (0%).

Specific siRNA-mediated suppression of FREP3 expression in BS-90 snails was confirmed at both the transcriptional (**Fig. 1A**) and protein levels (**Fig. 1B**) using RT-PCR and Western blot respectively. To assess whether FREP3 participated in an anti-*S. mansoni* defense response a total of 70 *S. mansoni*-resistant BS-90 strain snails were injected with FREP3-specific siRNA oligos to assess the impact of FREP3 knockdown on the subsequent ability of *S. mansoni* to develop. Knockdown of FREP3 resulted in cercariae-producing *S. mansoni* infections in 15 (21.4%) of these normally resistant snails (**Fig. 1C**). In contrast, none of 64 control BS-90 snails receiving GFP specific siRNA oligos shed cercariae. As a check of the viability of the *S. mansoni* miracidia used in this experiment, over 85% of schistosome-susceptible M-line snails exposed to infection in both trials became infected, a level of infection typical for exposure of such snails (**Fig. 1C**).

### Histological analysis of FREP3 knockdown BS-90 snails positive for *S. mansoni* infection

**Figure 2:**
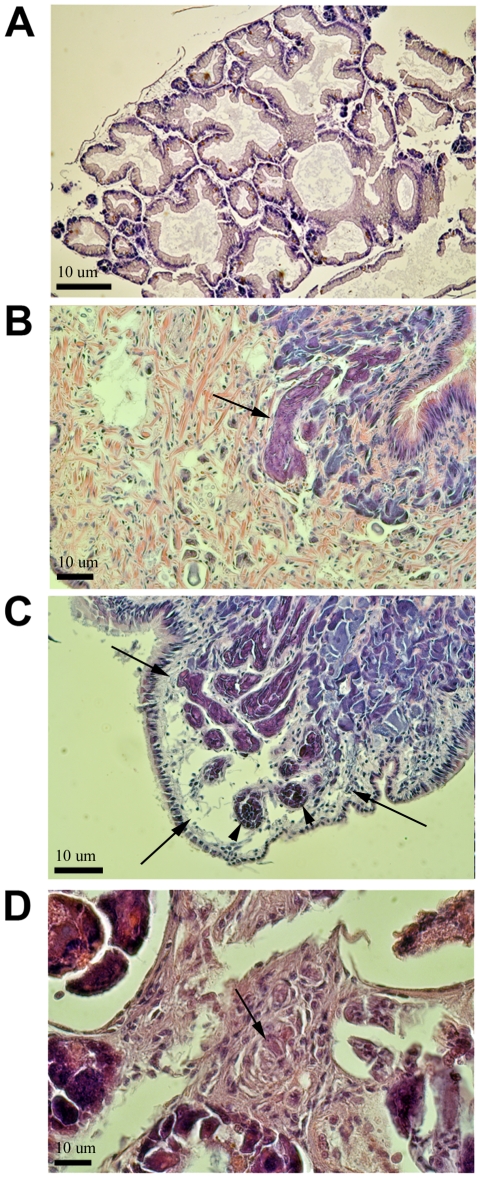
Histological sections of BS-90 strain *B. glabrata* snails treated with siRNA oligos targeting FREP3, at 31 dpe to *S. mansoni*. A. Snail digestive gland showing lack of *S. mansoni* infection in this organ. B. Enlarged sporocyst (arrow) in the head-foot. C. Another example from a different snail of enlarged, head-foot sporocyst (large arrows) showing germ balls (short arrows). D. Hemocyte reaction of snail to sporocyst material in the digestive gland. Scale bar = 10 um.

Histological observations revealed that *S. mansoni* miracidia penetrated snails receiving either FREP3 or GFP siRNA oligos. The early stage mother sporocysts (from 2 to 4 days post-infection) we observed were not conspicuously encapsulated in either group of snails. In most of the FREP3 knockdown snails that shed cercariae, shedding was light and intermittent over a 1–2 week observation period, after which they were fixed for histology at 31 days post-exposure to *S. mansoni*. Histological examination of *S. mansoni*-challenged FREP3 knockdown BS-90 snails revealed a small number of large sporocysts in the head-foot of each of these snails (**Fig. 2 B, C**). No disseminated daughter sporocysts were found in the digestive glands of these snails, however (**Fig, 2A**). The head-foot sporocysts had clearly grown considerably in size beyond that of young mother sporocysts, and whether they represented mother, or ectopic daughter sporocysts could not be determined. They were not encapsulated by hemocytes, nor were hemocytes prominently found near them. Developing cercariae were not seen within them but the sporocysts were of a size that easily could have supported cercariae development.

One of the infected FREP3 knock-down snails more persistently released cercariae over a 2 week observation period. Histological examination revealed this snail to have daughter sporocysts disseminated throughout the digestive gland. Hemocytes were conspicuous around them and encapsulation responses were noted (**Fig. 2D**). Only one of eight control BS-90 snails injected with GFP-specific siRNA oligos and sectioned at 28 days post-exposure to *S. mansoni* was observed to contain *S. mansoni* sporocysts, but they had not grown and did not contain germ balls.

### Immunosuppression of the snail defense response by *E. paraensei* makes BS-90 snails permissive to *S. mansoni* infection

**Figure 3:**
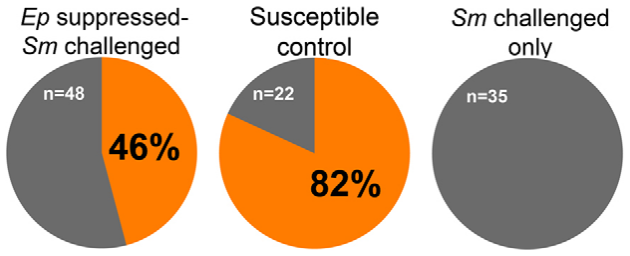
Percentage of BS-90 snails shedding *S. mansoni* cercariae after attenuation with irradiated *E. paraensei* miracidia. Experimental snails (46% infection rate) were compared to *S. mansoni*-susceptible M-line *B. glabrata* (82% infected), and to BS-90 snails challenged with *S. mansoni* only (0% infected).

**Figure 4:**
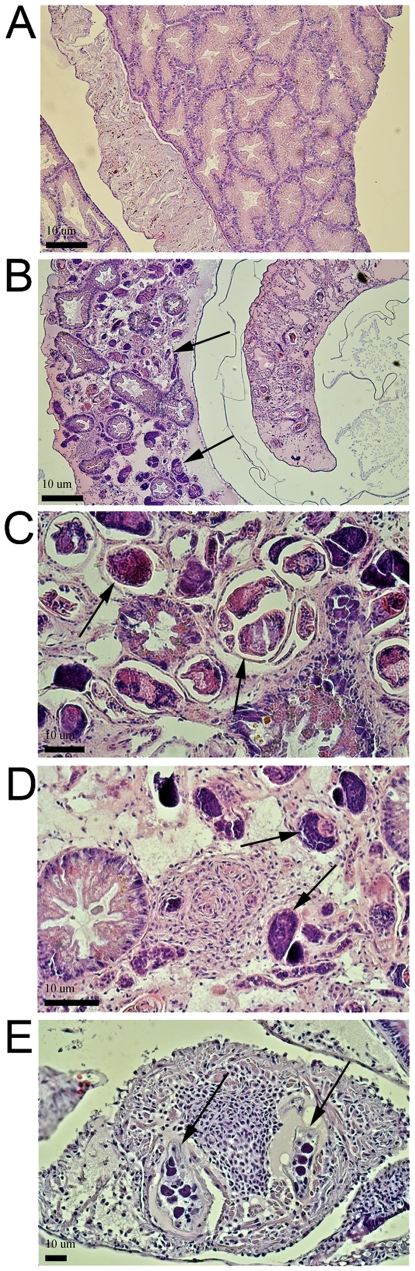
Histological sections of BS-90 snails made susceptible to *S. mansoni* infection by previous infection with irradiation-attenuated *E. paraensei*. A. Control snail not exposed to *S. mansoni* infection showing normal architecture of digestive gland. B–D. BS-90 snails with disseminated *S. mansoni* infections (arrows) following exposure to irradiated *E. paraensei*, including in the digestive gland. E. Exposure to irradiated *E. paraensei* did not result in disseminated infection or shedding of *E. paraensei* cercariae, however degenerating irradiated *E. paraensei* sporocysts were observed in the heart, as expected. Two degenerating sporocysts (arrows) of *E. paraensei* in the heart of a sensitized snail (28 dpe to *S. mansoni*). Scale bar = 10 um.

BS-90 snails exposed to irradiated *E. paraensei* miracidia were challenged with *S. mansoni* miracidia 4 days later. After another 35 days, the snails were checked for shedding of viable *S. mansoni* cercariae, an indication that the infection was successful. Of 48 snails, 22 (46%) shed *S. mansoni* cercariae, compared to 0% (n = 35) of control BS-90 snails exposed to only *S. mansoni* (**Fig. 3**). To confirm the infectivity of the *S. mansoni* used, 22 M-line *B. glabrata* were challenged and 82% were successfully infected (**Fig. 3**). Histological comparison of *S. mansoni* cercariae-shedding BS-90 snails to normal resistant control BS-90 snails (**Fig. 4A**) showed they had disseminated *S. mansoni* sporocysts throughout the digestive gland (**Fig. 4B, C**) typical of normal infections. Snails exposed to irradiation-attenuated *E. paraensei* only did not develop disseminated *E. paraensei* infections, as expected. Degenerating *E. paraensei* sporocysts could be observed in the hearts of the sensitized snails, including those subsequently challenged with *S. mansoni* (**Fig. 4E**). To confirm the viability of the *E. paraensei* cohort used, BS-90 snails were exposed to non-irradiated control miracidia from the same cohort that was irradiated and were successfully infected by 28 dpe, as expected (not shown).

### Successful infection of BS-90 snails by *S. mansoni* perpetuates the suppression of important defense factors

**Figure 5:**
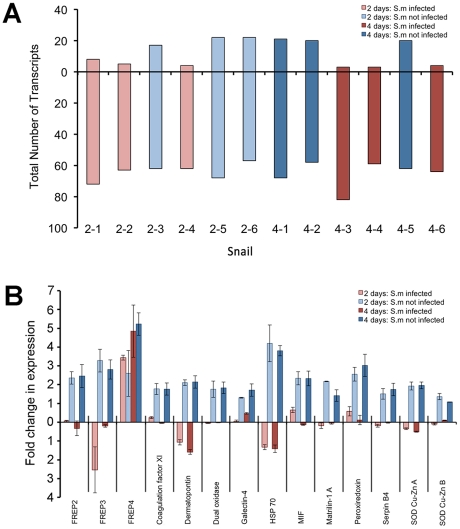
A. Total number of transcripts exhibiting increased (above zero line) or decreased (below zero line) expression in BS-90 snails immunocompromised by irradiated *E. paraensei* before challenge with *S. mansoni* (see key for bar colors on figure). Analysis compared experimental snails at 2 or 4 days post *S. mansoni* challenge to time and size matched control BS-90 snails exposed to irradiated *E. paraensei* only. Of the 6 individual snails analyzed at each time point, 3 were successfully infected with *S. mansoni*, and 3 remained resistant. B) Expression profiles of transcripts deemed important to *S. mansoni* resistance in snails. Fold-changes in expression of snails suppressed by irradiated *E. paraensei* before *S. mansoni* challenge are compared to snails exposed only to irradiated *E. paraensei*. Bars represent standard error (n = 3).

BS-90 snails first exposed to irradiated *E. paraensei* miracidia were challenged four days later with *S. mansoni* miracidia. Microarray analysis was then undertaken on individual snails either 2 or 4 days post-exposure to *S. mansoni*. Schistosome-specific markers on the array were used to indicate whether each snail had been successfully infected with *S. mansoni*, or if it had resisted the challenge. At both 2 and 4 days post *S. mansoni* challenge, 50% of the snails assayed using the array were positive for *S. mansoni* infections (3 positive, and 3 negative for *S. mansoni* for each time point). Immunosuppression (as indicated by the greater number of down-regulated than up-regulated features) resulting from exposure to irradiated *E. paraensei* miracidia was noticeable for all 12 snails studied with the arrays (**Fig. 5A**).

However, snails negative for *S. mansoni* markers displayed increased expression of a variety of known and putative defense-related factors (**Fig. 5B**). For some factors (FREP3, Dermatopontin, Heat shock protein 70, Superoxide dismutase 1 Cu-ZnA, Serpin B4, and Matrilin-1A) increased expression in snails negative for *S. mansoni* was contrasted by a suppression of expression in snails positive for this parasite. Other factors (FREP2, Coagulation factor XI, Dual oxidase, Galectin 4, Migration inhibition factor, Peroxiredoxin, and SOD Cu-Zn B) increased in expression in snails not infected by *S. mansoni*, but remained unaltered as compared to control values in snails that were successfully infected. FREP4 expression differed from other putative resistance molecules in that it was increased compared to control levels in both snails positive or negative for *S. mansoni* (**Fig. 5B**).

## Discussion

Schistosome parasites, including those that infect people, continue to thrive the world over, in no small measure owing their success to their productive use of snails as intermediate hosts. Particularly given that schistosome infection is harmful to the snail and results in its castration [18], it is reasonable to expect that the snail would mount defense responses to prevent infection. Although schistosomes obviously frequently prevail and establish long-term, infections, it is likely that many schistosome-snail encounters in the field result in failed infections. Such failures go overlooked but may well have a significant impact on transmission. Furthermore, the efficacy of present-day chemotherapy-based control operations could potentially be enhanced if we could also exploit snail resistance responses to further limit the number of new snail infections that arise. After all, it is in snails where cercariae - the source of reinfections in people that so frustrate control efforts - are produced in such prodigious numbers. To fully understand the potential impact of snail defenses on schistosome transmission to people, we need to achieve a better understanding of the mechanistic basis of snail defenses to infection, and how these defenses are overcome by schistosomes. In the process, we will also learn a great deal about the general nature of invertebrate (snail) defense mechanisms and the intimate interplay between host and parasite.

With respect to the immune responses of snails, our studies have lead us to focus on fibrinogen related proteins, or FREPs. One of the most noteworthy aspects of their biology is that two FREPs (first shown for FREP3, then FREP2) have been shown to undergo somatic diversification driven by gene conversion events and point mutations, creating a diversity of expressed sequences from a limited number of germ-line source sequences [10], [11], [19]. Recently, functional assessment of FREP3 demonstrated that it is capable of binding to carbohydrates and acts as an opsonin to enhance phagocytosis of targets by snail hemocytes. RNAi-mediated knockdown of FREP3 in snails resistant to the digenetic trematode *Echinostoma paraensei* resulted in an abrogation of resistance, resulting in one third of the snails developing established *E. paraensei* infections. Additionally, this study identified that FREP3, while increased in expression in resistant snails challenged with *S. mansoni* or *E. paraensei*, was suppressed in snails that were successfully infected by either parasite [11]. FREP2, another FREP that has the capacity for diversification, has been co-immuno-precipitated with *S. mansoni* polymorphic mucins, suggesting that this complex family of diversified parasite molecules may be the targets for FREPs [10].

Building on these earlier studies, here we demonstrate that FREP3 also plays a role in defense against *S. mansoni* infection. Knockdown of FREP3 resulted in 21% of the resistant BS-90 strain *B. glabrata* snails becoming successfully infected (shedding cercariae) with *S. mansoni*. In contrast, none of the 64 snails injected with GFP siRNAs shed cercariae. As previously hypothesized, FREP3 is likely working in combination with other defense mechanisms to manifest the resistant qualities of the BS-90 snails. However, this study clearly demonstrates that it is an important component of defense against *S. mansoni*.

Examination of sectioned snails revealed that *S. mansoni* miracidia penetrated both control GFP and experimental FREP3 knockdown snails, but observations of sporocysts at 2 and 8-days post-infection did not yield obvious evidence in either group of snails of sporocysts under conspicuous attack by hemocytes including within multilayered hemocyte capsules. Rather, sporocysts were found with only loose aggregates of hemocytes in their vicinity. This is compatible with observations reported by Galvan et al., 2000 [20] who noted that mother sporocysts of *S. mansoni* could remain viable in BS-90 snails for as long as 33 days. However, none of the *S. mansoni*-exposed BS-90 snails that they observed, nor any that we have observed over the years prior to this study, have ever shed cercariae. Our observations suggest that the inability of *S. mansoni* to thrive in BS-90 snails - at least in some cases - may be more dependent on inhibitory humoral factors than on overt hemocyte aggression and dismemberment. For example, humoral factors might serve to inhibit *S. mansoni* larval development or nutrition acquisition.

In all but one of the BS-90 FREP3 knockdown snails from which cercariae were shed, cercariae production must have originated from a small number of sporocysts in the head-foot of the snail. This likely explains why cercariae were produced by them in small numbers and intermittently. The daughter sporocysts producing these cercariae were either within or adjacent to the mother sporocyst that produced them. As these snails were fixed for histology, it is not clear how long they might have persisted in shedding cercariae. We suggest FREP3 knockdown in these snails allowed sporocysts to persist and enlarge, but was insufficient to enable them to proliferate and establish disseminated infections in the digestive gland. Hemocytes were not prominent around the head-foot sporocysts suggesting they had acquired some ability to protect themselves from attack. In snails receiving GFP siRNAs, in only one snail examined could sporocysts be found. They were small and showed no evidence of germ ball development. Based on these results, one possibility is that FREP3 plays a role in suppressing development of *S. mansoni* sporocysts in BS-90 snails, and if its effects are temporarily reduced, sporocysts may be released from this inhibition sufficiently well to enable some sporocyst development and multiplication to occur. As the knock-down effects inevitably wane, then the sporocysts may be prevented from further development such that proliferative infections do not usually result.

For the one FREP3 knockdown snail noted to have a disseminated infection, hemocytes accumulated in the digestive gland and in some cases were seen to be encapsulating daughter sporocysts. This is reminiscent of what was noted by Lie *et al.* [21] in some of the 10-R2 *B. glabrata* snails they observed in which resistance to *S. mansoni* had been broken down by pre-exposure of these snails to irradiated miracidia of *E. paraensei*. In about 30% of these snails, “self-cure” was eventually noted, characterized by hemocyte reactions to daughter sporocysts. Results of both experiments imply that snails inherently resistant to *S. mansoni* can reinvigorate an effective resistance response later in the course of infection, even though their ability to prevent establishment and development of infection had been earlier compromised by experimental manipulation. This suggests that the machinery for generating resistance is still intact. Furthermore, even though their collective biomass is large, daughter sporocysts may not be as effective as newly-penetrated (and much smaller) mother sporocysts in preventing effective responses.

As noted in the previous paragraph, and initially documented in studies by Lie and co-workers (13,25), both normal and irradiated sporocysts of *E. paraensei* have a potent ability to interfere with the resistance of *B. glabrata* to trematode infection. Their classic work has since stimulated a number of studies to reveal the underlying mechanisms of immunosuppression. Hemocytes collected from *B. glabrata* infected with *E. paraensei* exhibited reduced adhesive, spreading and phagocytic capacity compared to uninfected controls [22], [23]. *B. glabrata* hemocytes exposed to live *E. paraensei* sporocysts *in vitro* actively move away from the parasite [12], and eventually lose adherence to the substrate if exposed to parasite excretory/secretory (ES) products [24]. Furthermore, (ES) products of a related parasite, *Echinostoma caproni*, significantly impact the functional capacity and behavior of snail hemocytes, including a loss of adhesion, spreading and phagocytosis [25]. As these effects seem to be specific to suitable snail hosts, not extending to echinostome-resistant snail strains [25] or species [12], [26], the mechanism of their effects must be tailored to specific aspects of the defense system of compatible snails.

To pursue the molecular basis of *E. paraensei*-induced immunosuppression, we have followed the transcriptional responses of exposed snails using microarrays. By as early as 12 hours post-exposure, *E. paraensei* provokes down-regulation of snail defense responses, including FREP3 expression [7]. Many of the targets of *E. paraensei* immunosuppression are putative or known resistance-associated factors such as FREPs 1, 3, 5, 8, 9, and 10 [8], migration inhibition factor [27], dermatopontin [28], alpha-2- macroglobulin receptor [29], mannose receptor [30], peroxiredoxin [31], and galectins 4 and 7 [32]. Reduction in the presence of these factors theoretically would impact many aspects of defense function such as activation and phagocytosis by hemocytes (FREPs, mannose receptor, alpha-2- macroglobulin receptor), intra- and extra-cellular killing (peroxiredoxin), hemocyte adhesion and encapsulation (dermatopontin, migration inhibition factor), and coagulation (galectins).

Based on these results, we sought to repeat the basic design of the experiment of Lie et al. [14], to see if we could use pre-exposure of irradiated miracidia of *E. paraensei* to interfere with resistance of BS-90 snails to *S. mansoni*. Our experiment represents the first repeat of their classic experiment that has been accompanied by molecular (microarray) studies, and it is the first experiment to employ the naturally resistant BS-90 snails as hosts as opposed to other resistant *B. glabrata* snails of the 10-R2 or 13-16-R1 strains that were bred and selected for resistance [14].

We show that irradiated *E. paraensei* sporocysts suppress the BS-90 defense response sufficiently to allow 46% of the snails so treated to develop patent *S. mansoni* infections. When snails that permitted *S. mansoni* development were compared with those that did not using the *B. glabrata* microarray, we observed a number of immune-relevant transcripts that exhibited expression patterns indicating *S. mansoni* contributed to the suppression as well. FREP 2 and 3, coagulation factor IX, dermatopontin, dual oxidase, galectin 4, MIF, peroxiredoxin, superoxide dismutase Cu-Zn, and heat-shock protein 70 all exhibited increased expression in snails that successfully resisted infection compared to those that were infected by *S. mansoni*. Thus, we confirm previous hypotheses [7] suggesting that *S. mansoni* also utilizes a program targeted at suppressing the expression of important defense factors involved in killing larval parasites. This past work indicates that *S. mansoni* and *E. paraensei* differ in the timing and targets suppressed, *E. paraensei* beginning aggressive immunosuppression by 12 hours post challenge, *S. mansoni* beginning between 2 and 4 days post challenge [7].

Our results also suggest that irradiated echinostome larvae are more effective than our FREP3 knockdown protocol in protecting *S mansoni* sporocysts in resistant snails. This may be because irradiated echinostomes provide more persistent down-regulation of FREP3, and also have effects on other immune factors as well. The irradiated echinostome experiment indicates that if *S. mansoni* sporocysts are sufficiently protected, they can reliably develop disseminated infections in resistant snails. This may be because irradiated echinostomes provide *S. mansoni* sporocysts a longer interval to acquire and express their own immunosuppressive effects.

Our studies indicate that both echinostomes and schistosomes employ means of immunosuppression to colonize snails, and that this property can be manipulated to increase the breadth of strains of a single species, *B. glabrata*, that can be colonized. This work also bears on two important related general issues in parasitology, host specificity and host switching. Even though most digenetic trematodes are very host specific with respect to their choice of snail hosts, phylogenetic studies suggest that host-switching with respect to snails has been common in the history of trematodes like schistosomes [33]. The suppression we document offers one potential mechanism to resolve this apparent paradox: down-regulation of defense responses by one parasite may open the door for colonization of another parasite normally incompatible with that host. Field studies indicating the ability of one trematode to facilitate infection with another are consistent with this possibility [34], [35]. Cercariae produced in this study, both from BS-90 *B. glabrata* infected by *S. mansoni* due to reduced FREP3, or *E. paraensei*-mediated immunosuppression, were viable and able to infect mice. Thus, there is the potential for continuation of a trematode life cycle from a normally resistant snail host. It remains to be seen whether eggs produced from these mice have improved success at infecting BS-90 *B. glabrata*. We suggest that this study provides proof of principle that parasite-induced immunosuppression improves the chances that normally incompatible parasites can be successful in new, and hostile host environments. Furthermore, it provides a specific mechanism and molecules to target for future studies aimed at experimentally studying host specificity and host switching.

Another potential application of this work relates to the role of FREP3 in resistance of wild *B. glabrata* to infection with *S. mansoni*. Although it is clear that other factors are involved in resistance, this line of work suggests efforts to up-regulate FREP3 expression in snails from natural populations could have the effect of diminishing *S. mansoni* infections. We now must focus our efforts on understanding whether snails from endemic areas mount FREP3 responses following exposure to natural schistosome infections. It also raises the question as to whether snails differ in their inherent FREP3 responsiveness, and if this trait can be manipulated or favored to diminish natural schistosome infections.

## Supporting Information

Figure S1 Graph showing the fold change in expression of the transcripts observed to have altered expression patterns following knockdown of FREP3. A number of random transcripts (shown on the right side of the graph, separated by the vertical bar) are also shown to demonstrate the expression patterns observed for the majority of the transcripts on the array.
(TIF)
