# Supplementary figures and images for: A Somatically Diversified Defense Factor, FREP3, Is a Determinant of Snail Resistance to Schistosome Infection
Source: PLoS Negl Trop Dis. 2012 Mar 27;6(3):e1591. doi: 10.1371/journal.pntd.0001591 (PMC3313920; doi:10.1371/journal.pntd.0001591)

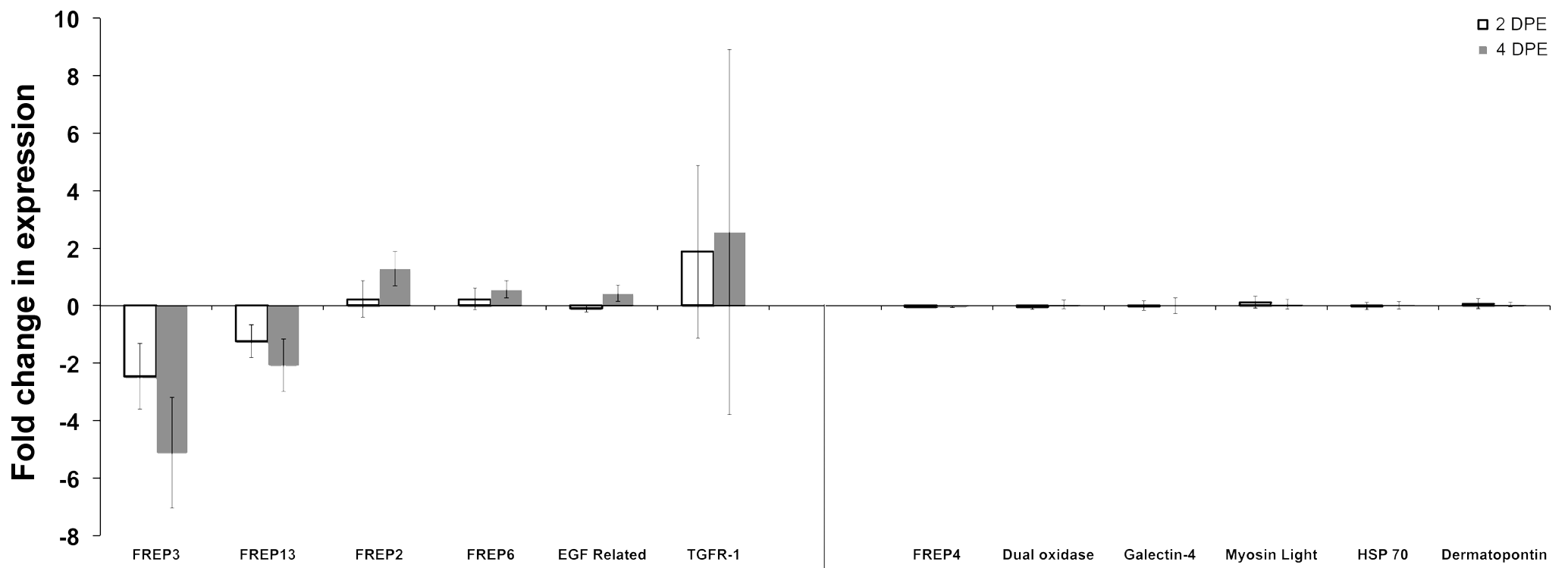

Supplement: Figure S1 — Graph showing the fold change in expression of the transcripts observed to have altered expression patterns following knockdown of FREP3. A number of random transcripts (shown on the right side of the graph, separated by the vertical bar) are also shown to demonstrate the expression patterns observed for the majority of the transcripts on the array. (TIF) [file pntd.0001591.s001.tif]
